# Valproate Damaging Effect on Erythrocyte Metabolism as a Decisive Factor in the Development of Encephalopathy

**DOI:** 10.3390/biom15040588

**Published:** 2025-04-15

**Authors:** Lyudmila Tikhonova, Eugene Maevsky, Carmina Montoliu, Elena Kosenko

**Affiliations:** 1 Institute of Theoretical and Experimental Biophysics of Russian Academy of Sciences, 142290 Pushchino, Russia; ljudasik09@rambler.ru (L.T.); eim11@mail.ru (E.M.); 2 Hospital Clinico Research Foundation, INCLIVA Health Research Institute, 46010 Valencia, Spain; 3 Pathology Department, Faculty of Medicine, University of Valencia, 46010 Valencia, Spain

**Keywords:** valproate, erythrocytes, glycolysis and PPP, redox signaling and oxidative stress, hypoperfusion, encephalopathy without liver failure

## Abstract

Background: Valproic acid (VPA) is a mainstay of treatment for epilepsy. Although VPA is generally considered well tolerated, it has serious adverse effects related to the pathological impact on cerebral perfusion and oxidative metabolism, leading to progressive encephalopathy. Erythrocytes directly deliver oxygen to the tissues. To understand how the brain pathology may be related to limited oxygenation, it is important to determine whether VPA-related changes occur in the intracellular erythrocyte metabolism responsible for the oxygen transport function. Methods: To determine whether different therapeutic VPA doses affect major metabolic pathways in rat erythrocytes, the activity of rate-limiting enzymes and levels of metabolites of glycolysis, the Rapoport–Luebering shunt, the pentose phosphate pathway and the antioxidant systems were measured. Results: Our data showed that VPA-induced G6PD inhibition leads to profound oxidative stress, increased MetHb formation and decreased 2,3-DPG and ATP levels in erythrocytes that underlie the loss of their oxygen transport function, thus being a cause of a brain energy crisis that precedes encephalopathy. Conclusions: The measurement of parameters in metabolic pathways modulating the redox-signaling and oxygen-carrying capacity of erythrocytes is needed for further elucidation of complex mechanisms underlying VPA-induced brain hypoperfusion and encephalopathy.

## 1. Introduction

Valproic acid (VPA) is a mainstay of treatment for epilepsy and other neuropsychiatric disorders [1]. Although VPA is generally considered well tolerated [2], it has serious adverse effects with fatal consequences recognized in the past in a relatively small group of patients treated with VPA [3,4], which is now confirmed by numerous studies and statistics [5,6]. Currently, it is widely accepted that valproate-induced progressive encephalopathy (VIE) refers to serious drug-caused neurological adverse effects [7], the clinical manifestations of which vary greatly from minimal disturbances of the conscious state and behavior [8,9] to dementia and coma, often characterized by fatal outcomes [10,11,12].

Typically, the most common VIE occurs in patients regardless of their age when the ammonia level in the blood increases (hyperammonemia, HA) [13,14], resulting from the VPA-induced defects of ammonia detoxification via ureagenesis and glutamine synthesis in the liver [4,15]. Ammonia, accumulated in the blood up to a toxic level, rapidly reaches the brain, hyperactivates NMDA receptors [16] and, in combination with reduced cerebral microcirculation [17], may have deleterious effects on the brain energy metabolism [18,19], aerobic glucose oxidation [20] and oxidative phosphorylation, thereby decreasing ATP synthesis [19].

Furthermore, disturbance of the antioxidant defense system by ammonia contributes to the enhanced production of reactive oxygen and nitrogen species [21,22], leading to the occurrence of oxidative stress [23,24,25], and blocks glutamine synthetase and many other essential processes [26], which together underlie the occurrence of a brain energy crisis and encephalopathy [27]. Therefore, it is generally accepted that blood ammonia is the main neurotoxin that plays a key role in the pathogenesis of VIE [28].

According to the data available that support the «VPA→ liver disfunction→ HA→ encephalopathy» relationship, VPA and its metabolites induce HA because of several mechanisms [6]. The main mechanisms that lead to HA are thought to be VPA-induced inhibition of the activity of the urea cycle enzyme, mainly liver mitochondria carbamoylphosphate synthetase-1 (CPS-1) [29], and other processes in the membrane [30] and matrix [31] of the mitochondria, which lead to the deficiency of ATP required for urea synthesis [32,33].

However, conflicting results concerning the effect of VPA on hepatic CPS-1 [34,35,36] suggest that the relationship between the VPA-induced overload of the liver detoxification system and a progressive loss of neuronal function is not well understood and cannot be taken as a general mechanism of VIE. Indeed, if VPA inhibits CPS-1 (as is the case with any other inhibitor or inherited urea cycle disorders), HA develops and then the concentration of urea in the blood begins to decline markedly [37,38]. However, some studies on VPA-related HA have found that no such relationship exists. Specifically, in some patients with VIE, despite the HA, the blood urea levels remained within the normal limits [39,40,41]. These findings show that the toxic effect of VPA on brain cells as a result of the liver detoxification function might be wider than predicted in terms of elevated blood ammonia levels provoked by urea cycle disorder.

Really, VPA-induced hyperammonemic encephalopathy occurs more often in patients without liver disease [42,43,44]. Moreover, VPA causes encephalopathy in some patients with normal blood ammonia levels [45,46,47,48]. After the discontinuation of VPA, neurological symptoms disappear rapidly [49].

Overall, based on these results and the above-mentioned normal blood ammonia levels (as is the case with normal urea levels) in some patients with VIE, it is impossible to assert unequivocally that blood ammonia is the primary neurotoxin initiating the encephalopathic effects of VPA.

In this regard, it is worth noting that in normal physiology, ammonia, as an important intermediate of cellular metabolism [18], is formed constantly in the brain (as in all other body tissues) [50]. Consequently, its levels inside the cell are not only based on the quantity arriving with blood but also on the balance between ammonia-forming and ammonia-consuming enzyme systems, which are strictly regulated to prevent the accumulation of endogenous ammonia to toxic levels and to maintain its steady-state intracellular levels [51]. In pathological conditions associated or unassociated with HA and in some pharmacological interventions [52,53], the unexpected oxygen–glucose deprivation [54] and the decline in cerebral energy metabolism [55] may occur. As a result, the endogenous formation of ammonia in the brain cells dramatically increases and the neurotoxin can be released into the circulation regardless of the liver-detoxifying function [56,57].

VPA is known to also exert an adverse effect on the neuronal oxidative metabolism [31,58], so it can be assumed that this drug might impact ammonia homeostasis in the brain [59].

Obviously, to get closer to a more complete understanding of VIE, in conjunction with the available evidence, it is also necessary to take into account that the brain compared to other organs, to maintain cell viability and its myriad functions, has the highest level of oxidative metabolism [60]. Paradoxically, the brain has limited oxygen reserves [61] and therefore requires a continuous supply of oxygenated blood. In line with this, a limited oxygen supply to the brain even for a few seconds leads to brain damage [62,63] and loss of consciousness. At the same time, chronic oxygen deficiency (regardless of the etiology of the disease) can stipulate hypoperfusion, hypoxic brain injury and encephalopathy accompanied by coma, often resulting in potentially lethal outcomes [64,65,66], thereby indicating the development of a common metabolic disorder in the presence of various pathologies.

The studies that have investigated the VPA effect on cerebral blood flow (CBF) and local cerebral glucose metabolism in healthy volunteers [67] or in patients with epileptic seizure [58] showed that VPA treatments even in the lowest doses (less than 20 mg/kg bm) resulted in statistically significant reductions in global CBF and regional suppression of cerebral metabolism. The effect of higher therapeutic doses of VPA (200–1000 mg/kg body mass) that are commonly used for the treatment of epilepsy in adolescents and adults [68] on these parameters is currently unknown. Although it remains to be seen, it is obvious that if the drug reduces the CBF and suppresses the oxidative metabolism of the brain especially at doses 10–50 times lower than the therapeutic ones, it can then worsen the outcome of the disease [69,70]. And this fact is one of the convincing arguments for obtaining a prompt reply to the following question: why does VPA even in minimal doses limit the oxygen supply to the brain and what is the mechanism(s) of the drug-induced cerebral hypoperfusion leading to encephalopathy without liver failure and HA?

Transport of sufficient oxygen to tissues requires the coordinated action of the three major systems: cardiovascular and respiratory systems and erythrocytes, which directly carry the oxygen to the cells [71]. However, the search for mechanisms underlying the cerebral imbalance between oxygen supply and demand, which provokes brain energy crisis and encephalopathy, usually tends to determine the pathological factors responsible for the impairment of the interdependent relationship between the respiratory and cardiovascular systems. Importantly, erythrocytes, which play a major role in this system, are not taken into account at all. Unfortunately, erythrocytes are still viewed in a simplified way, namely, as the cells for the function of which the endogenous hemoglobin level is important, rather than the Hb ability to bind oxygen as much as possible in the lungs and release it in the required amount to tissues [72]. That simplistic vision of erythrocytes, however, misses the key point: the O_2_ function of erythrocytes depends on intracellular metabolites that regulate the Hb-oxygen affinity [73,74].

At the same time, metabolites and enzymes involved in intracellular metabolism, which are responsible for hemoglobin-O_2_ affinity, flexible disc shape and deformability [75], NO-dependent hypoxic vasodilation [76], tissue perfusion [77] and the oxygen transport function of erythrocytes in general, are ignored. This may lead to erroneous conclusions about the determination of the real reasons as to why tissue hypoxia develops and the detection of regional perfusion abnormalities, thereby contributing to the failure to achieve positive clinical outcomes.

VIE is not an exception. VPA is active when exposed to erythrocytes circulating in the bloodstream [78] and causes various disorders, such as the suppression of glucose transport associated with the downregulation of glucose transporter GLUT1 [79]. Also, methemoglobinemia [80], severe membrane damage [81], an increase in the mean corpuscular volume [82], changes in the cell morphology [81] and many other vital processes [12,83,84] that affect the ability of erythrocytes to carry oxygen result from the erythrotoxic effect of VPA. Nevertheless, the causal relationship between erythrocyte intracellular metabolism abnormalities and their disfunction, resulting in VIE, has not yet been established.

Therefore, our primary objective in this study was to identify the “missing link”, namely, to determine whether different therapeutic doses of VPA have an effect on the major metabolic/energy pathways in the rat erythrocyte activity of rate-limiting enzymes, and the metabolite levels in glycolysis, the Rapoport–Luebering shunt, the pentose phosphate pathway and the antioxidant systems were measured.

Thus, taking into consideration the relationship between metabolic alterations in the circulating erythrocytes causing impairment of the erythrocyte oxygen transport function and brain hypoperfusion, the identification and quantification of the endogenous biochemical parameters of erythrocytes are necessary for revealing risk factors in the development of an unfavorable prognosis of hypoxia-induced brain energy crisis and encephalopathy in patients treated with VPA.

## 2. Materials and Methods

### 2.1. Materials

All the chemical reagents used, VPA, Tris, HEPES, TEA, EGTA, EDTA, NAD^+^, NADH, NADP^+^, NADPH, ATP, ADP, EGTA, phosphoenolpyruvate, glucose, pyruvate, ouabain, saponin, myokinase, pyruvate kinase (PK), lactate dehydrogenase (LDH), glucose-6-phosphate dehydrogenase (G6PD), glyceraldehyde-3-phosphate dehydrogenase (GAPDH), glutamate dehydrogenase, xanthine oxidase, glutathione reductase (GR), catalase (CAT), 1-chloro-2,4-dinitrobenzene, alfa-cellulose and hemicrystalline cellulose type 50, were purchased from Sigma Chemical Co. (St. Louis, MO, USA). The 2,3-diphosphoglycerate (2,3-DPG) concentration was determined with a test kit (Roche, Austria). Other reagents were produced in Russia and were of ultra-high purity.

### 2.2. Experimental Section

#### 2.2.1. Experimental Design

This study was conducted in accordance with the ethical principles underlying the Declaration of Helsinki for animals as an ethical “best practice” in clinical veterinary research and the Regulations of the European Science Association (revised in the European Directive 86/609/EEC and formulated in the Order of the Ministry of Health of the Russian Federation of 19.06.2003.267 “Guidelines on good laboratory practice”).

#### 2.2.2. Animals

Male Wistar rats weighing 210–230 g were used. The animals were housed in the animal facility at room temperature (22 °C) under natural light conditions with free access to food and water. The rats were divided into six groups (one control and five VPA groups) with 10 animals in each group.

VPA is rapidly cleared from rat plasma and transferred into the brain within a few minutes after its administration [85], and its neurological adverse effects and severe HA may develop suddenly [86] even after the administration of both defined daily doses [87] or drug overdoses [88]. So, decapitation of the animals in the VPA groups was performed 30 min after intraperitoneal injection of VPA with different single doses of 50, 100, 250, 500 and 1000 mg/kg bm. The selection of VPA doses was based on evidence collected in animal studies [89]; the range for the drug was close to that used in the treatment of human status epilepticus [90].

The VPA was dissolved in a sterile solution of 0.9% NaCl at a concentration of 400 mg/mL, with pH = 7.5. The same injections but with an equal volume of saline were performed in the control animals that were then decapitated 30 min after injection. All the experiments were carried out at 10:00 am to avoid changes in enzyme activities and metabolite levels due to circadian variations.

#### 2.2.3. Preparative and Analytical Methods

The blood samples obtained after animal decapitation (130 mM trisodium citrate was used as anticoagulant; pH 7.4) were divided into two portions (portion 1 and portion 2). To obtain blood plasma (portion 1), the blood cells were sedimented by centrifugation at 1000× *g* for 10 min (4 °C) and the supernatant (plasma) was deproteinated (1:2) with a cold mixture (–20 °C) of 6% HClO_4_ and 40% ethanol and deacidified with 30% (*w*/*w*) KOH (–20 °C) up to pH 6. After precipitation of the KClO_4_ crystals, the mixture was centrifuged (1000× *g*, 10 min and 4 °C) and the resulting supernatant was used immediately to determine the content of glucose in the plasma.

The erythrocytes (blood of portion 2) were purified from leukocytes and platelets by column chromatography. For this purpose, blood was applied to a column packed with alpha-cellulose and hemicrystalline cellulose type 50 (1:1) and equilibrated with 0.9% NaCl. Elution was performed (1:5) at room temperature with a solution containing 10 mM KH_2_PO_4_, pH 7.4 and 150 mM NaCl. The erythrocytes were pelleted by centrifugation at 4 °C for 10 min at 1000× *g*; washed twice with 10 mM KH_2_PO_4_ (pH 7.4) containing 140 mM NaCl, 5 mM KCl and 0.5 mM K-EDTA (10 min, 4 °C, 1500 g and 2000 g); and resuspended in the same solution at a 1:5 ratio (*v*/*v*).

**Preparation of erythrocyte extracts for determination of concentration of metabolites.** The purified erythrocytes were mixed with a cooled mixture (–20 °C) of 6% HClO_4_/40% C_2_H_5_OH at a ratio of 1 to 10. The resulting solution was centrifuged at 4 °C for 5 min at 10,000× *g*. The pH value in the supernatant was adjusted to 5–6 using 30% (m/m) KOH and dry KHCO_3_. The precipitate of potassium perchlorate was removed by centrifugation under the same conditions. The clear supernatant solution was immediately employed for the determination of the concentrations of ATP, ADP, AMP, lactate, pyruvate and 2,3-diphosphoglycerate. The concentrations of the adenine nucleotides, lactate and pyruvate were measured by common spectrophotometry procedures [91] described in our previous study [92]. The energy charge (EnC) was calculated using the Atkinson’s equation [93] EnC = (ATP + 0.5ADP)/(ATP + ADP + AMP). The NAD^+^/NADH ratio was estimated by the method of Williamson et al. [94], based on the equilibrium constant of 1.11 × 10^−4^ for the lactate dehydrogenase reaction. The concentration of 2,3-DPG was measured spectrophotometrically with a commercial kit (Roche, cat. # 10 148 334 001) containing a mixture of enzymes (phosphoglycerate mutase, phosphoglycerate kinase, GAPDH, triosephosphate isomerase and glycerol-3-phosphate dehydrogenase) according to the enclosed instructions.

**Preparation of lysates from erythrocytes for determination of enzyme activity.** The erythrocyte samples (1 mL) purified from platelets and leukocytes were lysed in 2 mL of a hypoosmotic lysis buffer (50 mM TEA, pH 7.4/0.15 mM K-EGTA and 3 mM beta-mercaptoethanol) containing 0.2% saponin. Enzyme activity was determined within the first two hours following the preparation of the lysates. In the course of the measurements, the probes were stored at 4 °C.

**Enzymatic activity assays in erythrocytes**. The activity of the superoxide dismutase (SOD, EC 1.15.1) was measured spectrophotometrically by following a decrease in the rate of the p-nitrotetrazolium blue dye (NTB) reduction in the xanthine-xanthine oxidase system at 550 nm (25 °C) [95]. The amount of the enzyme required for 50% inhibition of the NTB reduction was defined as one unit of enzymatic activity. The activity of the CAT (EC 1.11.1.6) was estimated from the decrease in the absorbance at 240 nm in the reaction with hydrogen peroxide [96] and expressed as the rate constant for a first-order reaction (s^−1^ per g Hb). The activity of the glutathione peroxidase (GP, EC 1.11.1.9) was determined spectrophotometrically at 25 °C from the decrease in the absorbance at 340 nm caused by NADPH oxidation by GSSH [97]. The activity of the GR (EC 1.6.4.2) was measured spectrophotometrically at 340 nm from the rate of NADPH oxidation by GSSH [98]. The activity of the glutathione transferase (GT, EC 2.5.1.18) was estimated spectrophotometrically at 340 nm from the rate of GSH binding to 1-chloro-2,4-dinitrobenzene at 340 nm [99]. The activity of the G6PD (EC 1.1.1.49) was measured spectrophotometrically by following the increase in the absorbance at 340 nm caused by the NADP^+^ reduction at 37 °C [100]. The activities of the GP, GR, GT and G6PD were expressed as μmol/min × g Hb. The activities of the Na^+^/K^+^-ATP-ase pump (EC 3.6.3.9), hexokinase (HK, EC 2.7.1.1), phosphofructokinase (PFK, EC 2.7.1.11), PK (EC 2.7.1.40), GAPDH (EC 1.2.1.12) and LDH (EC 1.1.1.27) were determined spectrophotometrically by the rates of NAD^+^ or NADPH formation at 340 nm using the methods of enzymatic analysis for erythrocytes. All the indices of the blood plasma and erythrocytes were assayed by using the standardized methods developed by the International Council for Standardization in Hematology [101] and the methods described in *Methods of Enzymatic Analysis* [102].

#### 2.2.4. Statistical Analysis

The statistical analysis was performed with the Prizm V8 software package (GraphPad Software Inc., La Jolla, CA, USA). All the data were presented as the mean ± standard error of the mean (SEM). The normality of distribution of variables was confirmed by the Kolmogorov–Smirnov test. The differences between the groups were analyzed with the Student’s *t*-test; ANOVA with the Bonferroni correction was used for multiple comparisons.

## 3. Results

### 3.1. Effects of Different Doses of VPA on Glucose Concentration in Rat Plasma and Erythrocytes

**Figure 1:**
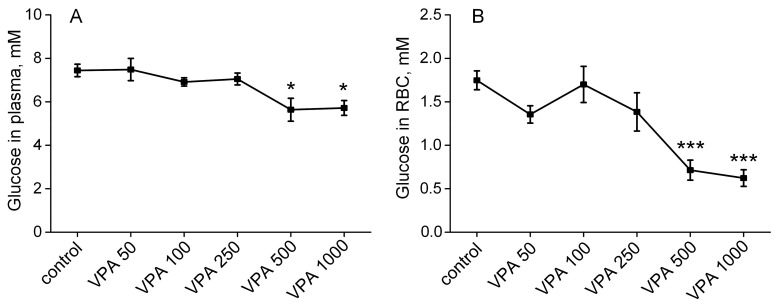
The effects of five doses (50, 100, 250, 500 and 1000 mg/kg bm) of VPA when administered intraperitoneally on glucose concentration in rat plasma (**A**) and erythrocytes (**B**). The animals in the VPA group received a single ip injection of different doses of VPA in 0.9% NaCl. The animals in the control group were given an equal volume of saline by the same route. The animals of both groups were decapitated 30 min after injection. The results are given as the mean ± SEM (*n* = 10 rats per group). The values marked with an asterisk are significantly different from those of the control group: * *p* < 0.05; *** *p* < 0.001 (with the Bonferroni correction for multiple comparisons).

A direct effect of VPA on erythrocyte glucose levels is still unknown so far. Moreover, plasma glucose studies in patients maintained on VPA monotherapy have yielded inconclusive results [103,104]. Therefore, to assess whether the impact of VPA in vivo on circulating erythrocytes could alter their energy metabolism, the effects of the drug on the glucose levels in rat plasma and erythrocytes were explored. The results are summarized in Figure 1.

Our findings demonstrate (Figure 1A) that the measured plasma glucose levels fluctuated within the normal limits in rats, receiving VPA with doses of 50–250 mg/kg bm, and did not differ significantly from those in the control group. Increasing the VPA dose to 500 mg/kg bm resulted in a significant decrease in the glucose concentration by 25% compared to the controls. However, a glucose level in the rat plasma remained equally low even when the VPA dose was increased up to 1000 mg/kg bm. The effect of high doses of VPA on the glucose level in erythrocytes was more pronounced than that in the plasma. It can be seen (Figure 1B) that VPA with doses up to 500 mg/kg bm did not cause a reliable change in the glucose level in erythrocytes (as in the plasma), but in doses of 500 and 1000 mg/kg bm, it led to a significant, twofold decrease in the glucose concentration.

These results indicate that high doses of VPA cause disturbances in blood glucose homeostasis in the animals. At the same time, it is obvious that if under these conditions the concentration of glucose in the plasma decreases by no more than 25% (Figure 1A), this is not enough to directly reduce the utilization of glucose by tissues and, in particular, by erythrocytes [105,106]. However, if over a short period of time (30 min) VPA with doses of 500 and 1000 mg/kg bm reduces the level of glucose in erythrocytes by half (Figure 1B), this indicates that the drug with these doses may have a direct effect on the glucose metabolism, which is critical for the maintenance of erythrocyte viability [73,74].

### 3.2. Effect of Different Doses of VPA on the Activity of the Regulatory Enzymes of Glycolysis in Rat Erythrocytes

**Figure 2:**
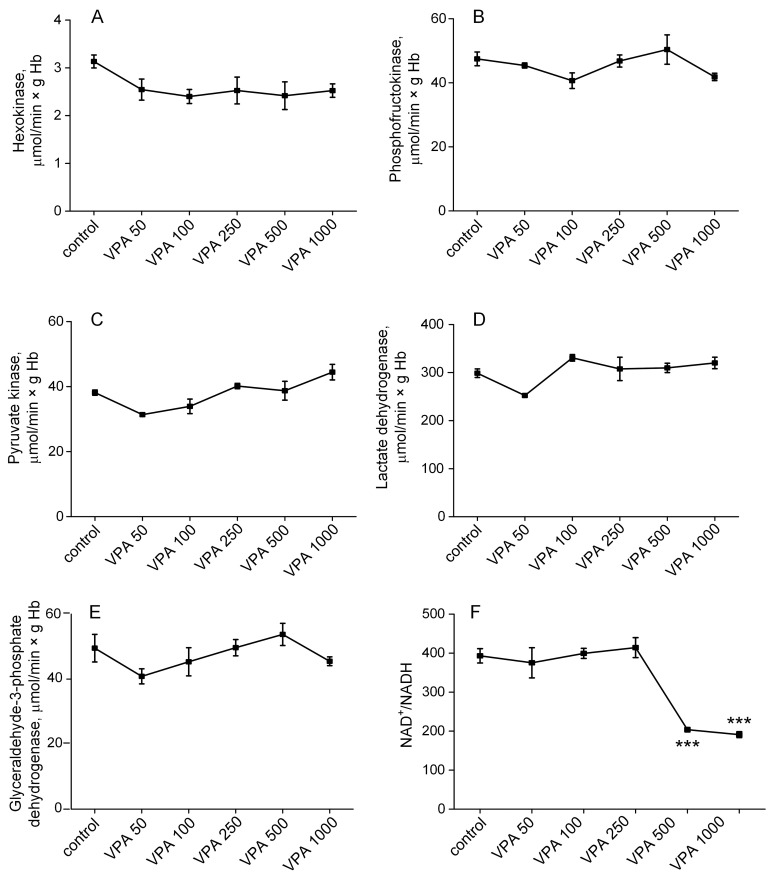
The effects of five doses (50, 100, 250, 500 and 1000 mg/kg bm) of VPA, when injected intraperitoneally, on the activity of regulatory enzymes of glycolysis hexokinase (**A**), phosphofructokinase (**B**), pyruvate kinase (**C**), lactate dehydrogenase (**D**), glyceraldehyde-3-phosphate dehydrogenase (**E**), and the NAD^+^/NADH ratio (**F**) in rat erythrocytes. Specific enzyme activities are expressed as μmol/min/g Hb. The animals in all the groups were decapitated 30 min after VPA or saline injection. The results are presented as the mean ± SEM (*n* = 10 rats per group). The values marked with an asterisk are significantly different from those of the control group: *** *p* < 0.001 (with the Bonferroni correction for multiple comparisons).

To test whether the limitation of erythrocyte glucose availability caused by high doses of VPA may affect the rate of glucose utilization, the activities of the major regulatory enzymes of the glycolytic pathway such as HK, PFK and PK as well as GAPDH and LDH were studied (Figure 2).

As can be seen (Figure 2), no significant differences in the activity of all the measured enzymes in the erythrocytes were observed between the groups of rats that received different doses of the drug and the control group. Thus, it can be assumed that glycolysis under the influence of all the studied doses of VPA is not limited by the activity of regulatory enzymes. In support of this, no significant differences were observed in the NAD^+^/NADH ratio between the control group and rats receiving VPA at a dose of 50 to 250 mg/kg bm, and only the highest doses of VPA (500 and 1000 mg/kg bm) contributed to a reduction (almost twofold) in the NAD^+^/NADH ratio as compared to the control (Figure 2). Since an unchanged and especially reduced NAD^+^/NADH ratio does not interfere with the process of NAD^+^ regeneration in the LDH reaction, and a glycolysis rate is reestablished at the stage of the GAPDH reaction [107], it can be reasonably assumed that although VPA, used at all the doses studied, inhibits glucose transport into erythrocytes [108], this drug has no significant effect on the rate of glycolytic flux even when the concentration of glucose in erythrocytes decreases by a factor of two but remains much greater than the Km values for glucose in the hexokinase reaction [109].

### 3.3. The Influence of Different Doses of VPA on the Content of Adenine Nucleotides in Rat Erythrocytes

The steady-state level of ATP depends not only on the rate of its formation but also on the activation of endogenous ATP-dependent reactions and on the release of ATP following exposure to β-adrenergic stimulation, reduced erythrocyte deformability and oxygen tension, or acidosis [110]. Therefore, the next step in our study was to examine whether VPA, which does not affect the glycolytic glucose pathway, can impact ATP and other adenine nucleotide levels in rat erythrocytes.

**Figure 3:**
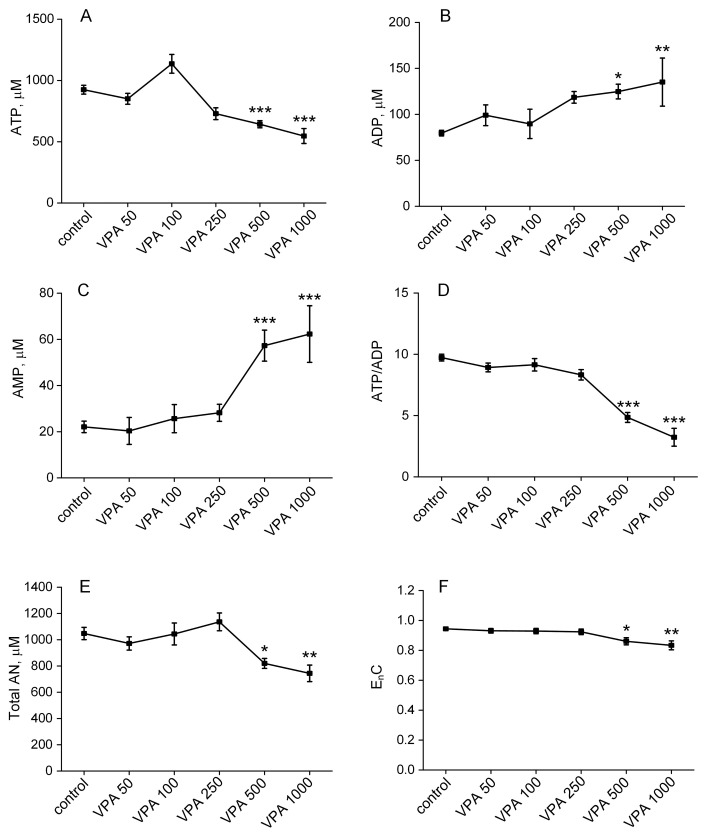
The effects of five doses (50, 100, 250, 500 and 1000 mg/kg bm) of VPA, when injected intraperitoneally, on the concentrations of ATP (**A**), ADP (**B**) and AMP (**C**); the ATP/ADP ratio (**D**); and the total amount of AN (**E**) and EnC (**F**) in rat erythrocytes from experiment groups (*n* = 10 per group). The concentration of metabolites is in µmol/L (µM). The energy charge was calculated according to Atkinson [93]: EnC = (ATP + 0.5ADP)/(ATP + ADP + AMP). The data are expressed as the mean ± SEM. * *p* < 0.05, ** *p* < 0.01 and *** *p* < 0.001 as compared to the control group of animals. Differences between the groups were explored using ANOVA followed by Bonferroni corrections.

Figure 3 represents the results obtained after the determination of the ATP, ADP and AMP concentrations and calculated values of the total adenine nucleotide content (AN), adenine nucleotide ratio (ATP/ADP) and EnC of the adenylate pool, which is a metric indicating the energetic status of the cell.

As can be seen from Figure 3, there was a statistically significant change in all the measured parameters only when the VPA doses increased from 500 to 1000 mg/kg bm. Thus, a single injection of VPA at doses up to 250 mg/kg bm did not affect the intracellular concentration of ATP, whereas at doses of the drug of 500 and 1000 mg/kg bm, the concentration of ATP in the animal erythrocytes significantly decreased by 31% (*p* < 0.001) and 41% (*p* < 0.001), respectively, compared to the control (Figure 3A). Increased ATP hydrolysis at these doses of VPA contributed to elevations of the ADP levels by 50% (*p* < 0.05) and 64% (*p* < 0.01), respectively, (Figure 3B), and AMP concentrations by 159% (*p* < 0.001) and 182% (*p* < 0.001), respectively, (Figure 3C), compared to the control.

Accordingly, the calculated indicators of adenine nucleotides also changed only with an increased dosage of VPA up to 500–1000 mg/kg bm. Thus, at the given doses of VPA (500 and 1000 mg/kg bm), the ratio of ATP/ADP (Figure 3D) in the animal erythrocytes decreased by 51% (*p* < 0.001) and 68% (*p* < 0.001), respectively; the AN decreased by 22% (*p* < 0.05) and 28% (*p* < 0.01), (Figure 3E), and the EnC decreased by 8% (*p* < 0.05) and 12% (*p* < 0.01), respectively, (Figure 3F), as compared to the control.

This evidence shows that only higher doses of VPA induced an increased cellular demand for ATP that was not compensated by the normal glycolytic flux, resulting in a distinct disruption of adenine nucleotide homeostasis and a significant decrease in intracellular ATP levels.

### 3.4. The Influence of Different Doses of VPA on the Activity of Na^+^, K^+^-ATPase in Rat Erythrocytes

Most of the energy of glycolysis, stored as ATP, is coupled to membrane-bound Na^+^, K^+^-ATPase [111], which is necessary for normal intercellular Na^+^, K^+^ homeostasis and erythrocyte function [112]. Knowing that VPA even at therapeutic dosages may cause inactivation of membrane-bound proteins [113,114], the next step in our study was to measure the activity of Na^+^, K^+^-ATPase in the rat erythrocytes of all the studied animals treated with different doses of VPA.

**Figure 4:**
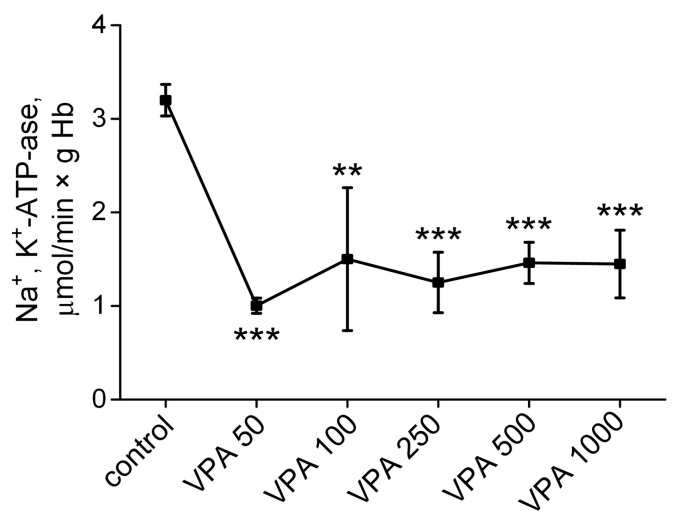
The effects of five doses of VPA (50, 100, 250, 500 and 1000 mg/kg bm), when administered intraperitoneally, on Na^+^, K^+^-ATPase activity in rat erythrocytes. The animals in all the groups were decapitated 30 min after VPA or saline injection. The data are expressed as the mean ± SEM (*n* = 10 rats per group). The values marked with an asterisk are significantly different from those of the control group: ** *p* < 0.01; *** *p* < 0.001 (with the Bonferroni correction for multiple comparisons).

The data are presentId in Figure 4.

As can be seen, 30 min after the injection of VPA at all the doses used, the activity of Na^+^, K^+^-ATPases in the rat erythrocytes sharply and almost equally (3 times) significantly decreased compared to the control. Our findings showed that enzyme activity began to decrease under the influence of low doses of VPA (50–250 mg/kg bm) when the intracellular ATP level remained unchanged compared to the control (Figure 3). These data indicate that the VPA-induced inhibition of Na^+^, K^+^-ATPase in these conditions does not depend exclusively on the availability of ATP, suggesting the activation of a specific VPA-related mechanism(s) as a switch toward the downregulation of this enzyme even at a VPA dose below 50 mg/kg bm.

### 3.5. Effect of VPA on the Activity of Antioxidant Defense Enzymes in Rat Erythrocytes

Reduced antioxidant enzyme activity results in the accumulation of reactive oxygen and nitrogen species (ROS) in the cells, making Na^+^, K^+^-ATPase vulnerable to oxidative stress [115]. VPA is thought to be a critical initiator of oxidative stress [116,117,118,119], and the activity of the enzymes of the antioxidant defense system similar to the activity of Na^+^, K^+^-ATPase is strongly and irreversibly suppressed in the presence of ROS [120,121]. So, the next step of our research was to determine whether VPA-induced suppression of Na^+^, K^+^-ATPase was associated with diminished activities of the main ROS-scavenging enzymes.

**Figure 5:**
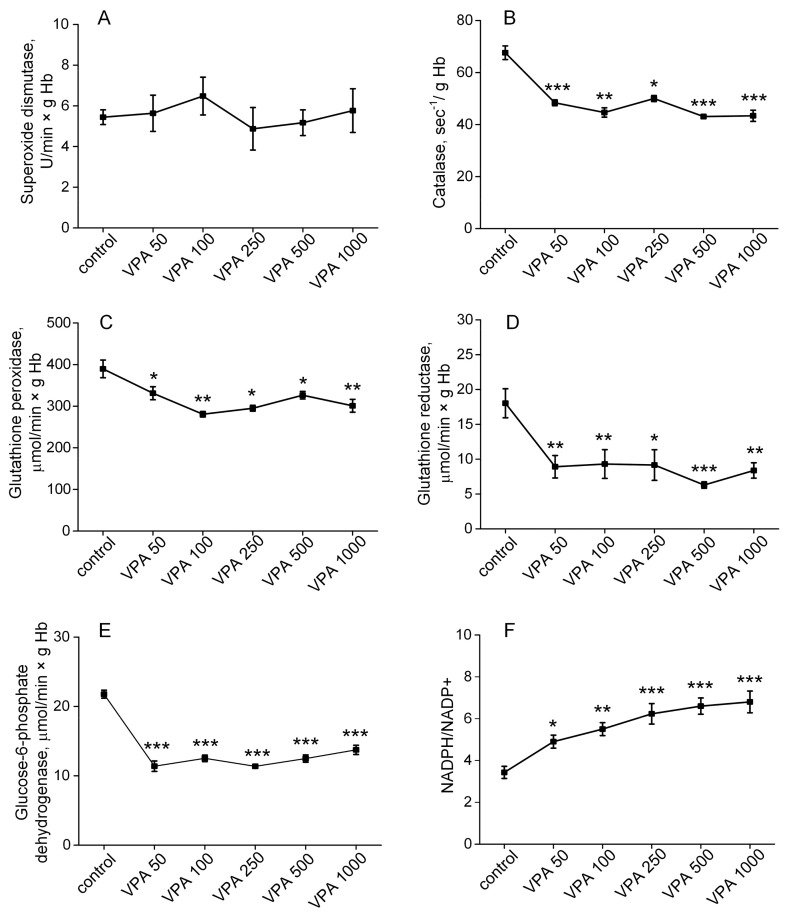
The effects of five doses of VPA (50, 100, 250, 500 and 1000 mg/kg bm), when administered intraperitoneally, on the superoxide dismutase (**A**), catalase (**B**), glutathione peroxidase (**C**), glutathione reductase (**D**), glucose-6-phosphate dehydrogenase (**E**) and the NADPH/NADP^+^ ratio (**F**) in the animal erythrocytes from all the studied groups. The NADPH/NADP^+^ ratio was calculated using the values of the reaction catalyzed by G6PD (NADPH/NADP^+^ = [glucose 6-phosphate]/[6-phosphogluconolacton]) [122] with the equilibrium constant of 1.3 [123]. The animals in all the groups were decapitated 30 min after VPA or saline injection. The data are expressed as the mean ± SEM (*n* = 10 rats per group). The values marked with an asterisk are significantly different from those of the control group: * *p* < 0.05, ** *p* < 0.01 and *** *p* < 0.001 (with the Bonferroni correction for multiple comparisons).

Figure 5 depicted the effects of different doses of VPA on the activity of SOD, CAT, GR, GP and G6PD in the animal erythrocytes from all the studied groups.

As seen, the activity of SOD in animal erythrocytes (Figure 5A) remained unaltered relative to the control values after the administration of VPA at all the doses used. On the contrary, the activity of CAT, GP, GR and G6PD significantly decreased under the influence of all the doses of the drug, but the degree of inhibition of the enzymes was different. Thus, a minimal decrease in CAT activity (28%, *p* < 0.001, Figure 5B) was observed when VPA was injected at a dose of 50 mg/kg bm, but a further increase in the VPA dose (100, 250, 500 and 1000 mg/kg bm) did not result in an additional decrease in the enzyme activity and it remained reduced to the same limit. A decrease in GP activity (17%, *p* < 0.05, Figure 5C) was observed when VPA was administered at a dose of 50 mg/kg bm. Increasing the VPA dose to 100 mg/kg bm reduced the GP activity by 26% (*p* < 0.01) when compared with the control, but a further increase in the VPA dose (250, 500 and 1000 mg/kg bm) did not result in a additional decrease in the enzyme activity. The decrease in GR activity was significantly more pronounced than a change in GP activity, but again, it was not related to the drug dose. It may be seen (Figure 5D) that the GR activity in the erythrocytes of the control animals, which was 18.03 ± 2.1 µmol/min × g Hb, significantly decreased to 8.9 ± 1.6 (*p* < 0.01) by the injection of VPA at a dose of 50 mg/kg bm and remained at the same low level with an increasing VPA dose of 1000 mg/kg bm.

The activity of G6PD, an enzyme of the pentose–phosphate pathway (PPP) that is not directly involved in ROS scavenging but provides the NADPH required both for GR- and catalase-mediated catabolism of H_2_O_2_ [124,125], also decreased almost twice in relation to the control under the action of the minimal (50 mg/kg bm) VPA dose, and there were no additional changes in the G6PD activity in response to higher doses of the drug (Figure 5E).

Together with the sharply increased NADPH/NADP^+^ ratio (Figure 5F), the results indicate that VPA, even at a minimal dose, significantly limits glucose metabolism through the PPP and that disruption of the intracellular NADPH redox balance through the inhibition of G6PD (Figure 5E) and the subsequent GR (Figure 5D), GP (Figure 5C) and Cat (Figure 5B) is tightly associated with VPA-induced increases in ROS formation [126,127]. This strongly suggests that the mechanism responsible for the decrease in Na^+^, K^+^-ATPase activity under the conditions used (Figure 4) (in addition to ATP deficiency) may involve a disturbance in redox homeostasis that makes the pump protein vulnerable to oxidative damage [115,120].

### 3.6. Methemoglobin Content in Rat Erythrocytes After Injection of VPA with Different Doses

**Figure 6:**
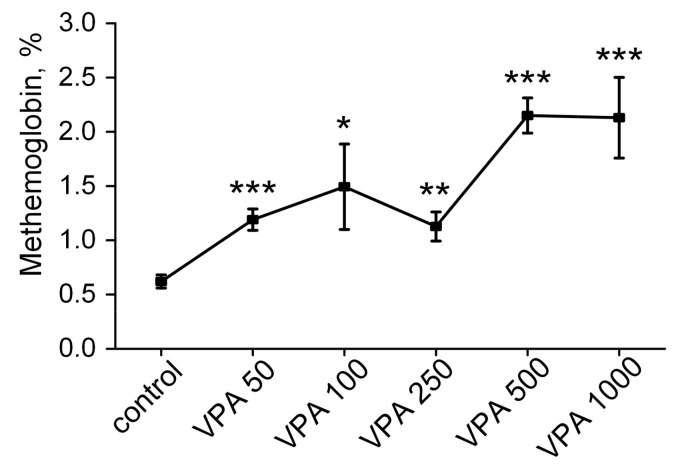
Effects of different VPA doses (50, 100, 250, 500 and 1000 mg/kg bm) on the methemoglobin levels (%) in rat erythrocytes. The animals in all the groups were decapitated 30 min after VPA or saline injection. The data are expressed as the mean ± SEM (*n* = 10 rats per group). The values marked with an asterisk are significantly different from those of the control group: * *p* < 0.05, ** *p* < 0.01 and *** *p* < 0.001 (with the Bonferroni correction for multiple comparisons).

G6PD deficiency is also the most common trigger for the formation of methemoglobin (MetHb), which is unable to bind to oxygen [128]. To determine additional indicators that are necessary for the assessment of the capability of erythrocytes to deliver oxygen to tissues, our study focused on an investigation of how a single administration of VPA at different doses may affect the MetHb level in rat erythrocytes (Figure 6).

As seen in Figure 6, the MetHb levels in the erythrocytes increased statistically significantly in all the VPA groups as compared to the control value of 0.62 ± 0.061%. A relatively small increase in the MetHb levels (up to 1.19 ± 0.09%; *p* < 0.001) was recorded in the erythrocytes of the rats receiving VPA at a dose of 50 mg/kg bm. The maximum increase in the MetHb level (2.15 ± 0.16%) was observed in the animals after the administration of VPA at a dose of 500 mg/kg bm (Figure 6) and there were no additional changes in the MetHb level in response to the higher drug dose of 1000 mg/kg. But while under the influence of these doses, the MetHb level was 3.5 times higher than the control value, and the threshold concentration of MetHb fell within the reference intervals usually given for the erythrocytes of rats kept under physiological conditions [129]. However, a faster trend toward elevation (30 min) in the MetHb level, in all the treated animal groups, seems to be a warning sign of inadequate tissue oxygenation since the effects of long-term usage of VPA during treatment on the MetHb level are not known.

### 3.7. The Effect of Different Doses of VPA on the Concentration of 2,3-DPG in Rat Erythrocytes

Another erythrocyte marker of impaired tissue oxygenation is 2,3-DPG, and its depletion in erythrocytes negatively affects the release of oxygen from the complex with Hb. Consequently, this impairs the transfer of oxygen to tissues [130].

**Figure 7:**
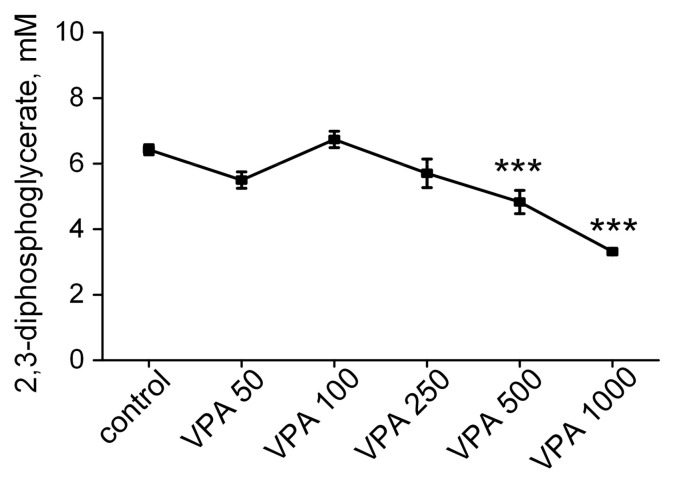
Effects of different VPA doses (50, 100, 250, 500 and 1000 mg/kg bm) on the 2,3-DPG level in rat erythrocytes. The requirements for drug administration, dosage and decapitation time are given in the legend to Figure 1. The concentration of the metabolite is in mmol/L (mM). The data are expressed as the mean ± SEM (*n* = 10 rats per group). The values marked with an asterisk are significantly different from those of the control group: *** *p* < 0.001 (with the Bonferroni correction for multiple comparisons).

The next step in our work was to find out how different doses of VPA impact the concentration of 2,3-DPG in rat erythrocytes. The essential data are presented in Figure 7. It can be seen that the level of 2,3-DPG in the erythrocytes of the animals that received VPA at doses of 50–250 mg/kg bm did not differ from the controls. With a VPA dose of 500 mg/kg bm, the level of 2,3-DPG depleted moderately (25%, *p* < 0.001, whereas with an increase in the dose of the drug to 1000 mg/kg bm, the level of 2,3-DPG decreased by 48% (*p* < 0.001) as compared to the control.

The observed trend toward a lower content of 2,3-DPG in the erythrocytes of the animals receiving VPA in doses of 500–1000 mg/kg bm is in agreement with the scientific evidence of the relationship between oxidative stress, methemoglobinemia and reduced 2,3-DPG levels in the erythrocytes. In addition, it indicates that VPA can simultaneously influence multiple processes that are directly implicated in the impairment of the capacity of erythrocytes to carry oxygen to tissues, leading to progressive hypoperfusion and hypoxia [131]. However, since the factors that regulate the steady-state level of 2,3-DPG in the erythrocytes are numerous and interrelated [132], it is clear that without specially conducted studies, it is impossible to determine what factors upon contact of VPA with circulating erythrocytes may affect the decrease in 2,3-DPG. And questions concerning the mechanism(s) underlying oxygen transport disorders by faster oxygen binding in the lungs and slower kinetics of its off-loading in tissues remain open.

## 4. Discussion

Despite the numerous reports that describe favorable effects of VPA as a highly effective anticonvulsant in the treatment of epileptic syndromes in children and adults [133], the expanded use of the drug to treat other mental illnesses, including bipolar, depression, anxiety, alcohol withdrawal and many others [134], has led to a steady increase in the number of cases reported of multi-organ failure [135,136] and, in particular, VIE [7], with often fatal outcomes [10,11,12,137]. The VIE etiology is unknown, but it is generally believed to be caused by the direct destructive effect of VPA on the ammonium-detoxifying function of the liver [29] and, therefore, blood ammonia is universally recognized as the major neurotoxin that plays a key role in the pathogenesis of VIE [28]. However, clinical and animal contradictory findings about the effect of VPA on liver function [34,35,36] as well as data showing that VPA causes encephalopathy in some patients with normal blood ammonia levels [45,46] indicate that the VPA impact on functional brain activity as a result of liver dysfunction may be wider than predicted based on increased blood ammonia due to urea cycle abnormalities.

Indeed, when considering that VPA, by reducing regional cerebral blood flow, depresses cerebral oxidative metabolism of glucose in patients with epileptic seizure [58,67], it can be understood that cerebral hypoperfusion, leading to brain energy crises and encephalopathy with declining mental status [138,139], is an integral part of the neurotoxic effect of VPA.

In addition, upon contact with the circulating erythrocytes, VPA exhibits pronounced erythrotoxicity, as viewed by deviations from the norm of the erythrocyte structure [82,140], underlying the impairment of rheological properties [141], the viscosity of blood and the effective microcirculation and, consequently, oxygen delivery to tissues, including reduced brain oxygenation [80], inevitably leading to brain pathology [61] and the progression of persistent cognitive dysfunction. Hence, it is evident that the erythrocytes are highly susceptible to VPA-induced injury and can provide early warning signals of abnormalities of tissue oxygenation and timely recognition of risk factors for the development of VIE. Surprisingly, this predictable pathological relationship at present remains practically unexplored.

In the current study, it was shown for the first time that the effect of VPA on metabolic pathways of circulating erythrocytes can already be observed in vivo 30 min after the drug administration, but it was not always dosage-dependent. Thus, it was found that VPA (up to 500 mg/kg bm) did not cause a reliable change in the level of glucose in erythrocytes (as in plasma) but in doses of 500 and 1000 mg/kg bm led to a significant decrease in glucose concentration by 59% (*p* < 0.002) and 65% (*p* < 0.004), respectively, as compared to the control (Figure 1).

These findings are consistent with the studies reporting that VPA significantly inhibits glucose transporter (GLUT 1) activity in erythrocytes in vitro [79] and suggests that the drug can be a driving force for numerous metabolic abnormalities that occur in glucose-deprived conditions in vivo. However, the exact threshold values of intracellular glucose at which the metabolism and functional capacity of erythrocytes become impaired in rats still remain disputable. Knowing that HK in rat erythrocytes has low Km values for glucose (less than 0.1 mM) [142], it can be assumed that the observed sharp decrease in the intracellular glucose concentration in rat erythrocytes under the influence of the maximum dose of VPA (1000 mg/kg bm) to 0.622 ± 0.096 mM should be sufficient (at least in the first 30 min after administration of the drug) to initiate the glycolytic pathway at the hexokinase step, even with VPA-induced low permeability for glucose.

Indeed, in cells deprived of glucose, no glycolytic enzymes whose activity differed from the control level were detected. VPA at any dosage did not affect the activities of HK, as well as other regulatory enzymes of glycolysis: PFK, PK, GAPDH and LDH (Figure 2). Additionally, the NAD^+^/NADH ratio was unchanged at a VPA dose of 50 to 250 mg/kg bm. Moreover, when the NAD^+^/NADH ratio decreased twofold relative to the control at the maximum VPA dose (Figure 2F), this generally indicated that VPA at all the doses studied did not interfere with the process of NAD^+^ regeneration in the LDH reaction and the restoration of glycolysis at the GAPDH reaction stage [107]. So, it can be suggested that VPA used at all dosages has no significant effect on the rate of glycolytic flux under conditions of the limited glucose transport into the cells.

However, after measurement of the concentration of ATP, the major macroerg produced by glycolysis, it was found that a single injection of VPA at doses less than 500 mg/kg did not affect the intracellular concentration of this metabolite. At the same time, while activities of glycolytic regulatory enzymes in the erythrocytes of the animals receiving all doses of VPA remained unchanged, a statistically significant decrease in the concentration of ATP by 31% and 41% was observed, only when VPA at doses of 500 and 1000 mg/kg bm was injected, respectively. (Figure 3A). Increased ATP hydrolysis under these high doses of the drug contributed to elevations of the ADP levels by 50% (*p* < 0.05) and 64% (*p* < 0.001), respectively, (Figure 3B), and of the AMP concentrations by 159% (*p* < 0.001) and 182% (*p* < 0.001), respectively, (Figure 3C), as compared to the control. Similarly, the calculated indicators of adenine nucleotides also changed only with an increased dosage of VPA up to 500–1000 mg/kg bm: the ATP/ADP ratio (Figure 3D) in the erythrocytes of the animals decreased relative to the control by 51% (*p* < 0.001) and 68% (*p* < 0.001), respectively; the AN decreased by 22% (*p* < 0.05) and 28% (*p* < 0.01), and the EnC decreased by 8% (*p* < 0.05) and 12% (*p* < 0.01), respectively.

This evidence shows that only higher doses of VPA induced an increased cellular demand for ATP that was not compensated by the normal glycolytic flux, resulting in a distinct disruption of adenine nucleotide homeostasis and a significant decrease in intracellular ATP levels. This is critical not only for the vital functions of the erythrocytes [143] but also for intercellular communication, which, for optimal O_2_ delivery and organ function, requires the release of ATP by erythrocytes to regulate blood flow [77] and tissue perfusion [110].

In addition, the breakdown of ATP into less energetically active phosphate compounds, ADP and AMP, and the dramatic accumulation of AMP in erythrocytes (Figure 3B,C) create the conditions for the activation of erythrocyte-specific AMP deaminase [144], which leads to significant intracellular formation and accumulation of ammonia, a powerful erythrotoxin, which in the absence of ammonia-detoxifying enzymes in erythrocytes, negatively affects energy metabolism, antioxidant potential, lifespan and cell function [145,146]. Furthermore, the subsequent release of ammonia accumulated in erythrocytes [147] stipulates an unpredictable and significant increase in the blood ammonia level (hyperammonemia), thereby provoking the occurrence of ammonium-induced encephalopathy [28].

Overall, the data obtained indicate that the effects of VPA on erythrocyte energy metabolism are multifactorial and indirect rather than direct. The identified erythrotoxic effects as in a case of a decrease in ATP (Figure 3A) may be a consequence of high doses (500–1000 mg/kg bm) that are usually used in clinical settings as normal therapeutic doses [148] of the drug.

Another indirect effect of VPA includes its effect on the activity of Na^+^, K^+^-ATPase, for which the maintenance of the most energy of glycolysis, stored as ATP, is needed. The dependency between ATP availability and the functional ability of Na^+^, K^+^-ATPase implies that if a statistically significant decrease in ATP concentration in animal erythrocytes is observed only with an increase in the VPA dose to 500–1000 mg/kg bm (Figure 3A), the activity of Na^+^, K^+^-ATPase in these cells has to decrease only under the influence of the same VPA doses.

However, it turned out that the activity of Na^+^, K^+^-ATPases in the rat erythrocytes sharply and almost equally (3 times) significantly decreased as compared to the control after the injection of VPA at all the doses used (Figure 4). It is noteworthy that the enzyme activity began to decrease under the influence of low doses of VPA (50–250 mg/kg) when the intracellular ATP content remained unaltered as compared to the control value (Figure 3). These data indicate that the VPA-induced inhibition of Na^+^, K^+^-ATPase under these conditions does not depend exclusively on the availability of ATP and additionally confirms the indirect effect of VPA on enzyme activity, which might conceivably be observed even at a VPA dose below 50 mg/kg bm.

Whereas the reasons of the enzyme inhibition are still unknown, our data suggest that VPA-induced oxidative stress in erythrocytes may be an extra cause (beyond the deficiency of ATP) of the global Na^+^, K^+^-ATPase inhibition. A major determinant triggering oxidative stress is G6PD, an enzyme of the pentose–phosphate pathway (PPP) that is not directly involved in ROS scavenging but provides the NADPH required of both the GR- and catalase-mediated catabolism of H_2_O_2_ [124,125]. In particular, our study showed that the activity of G6PD decreased almost twice in relation to the control under the influence of the minimal (50 mg/kg bm) VPA dose and was not further altered in response to higher doses of the drug. (Figure 5E). These observations and the sharply increased NADPH/NADP^+^ ratio (Figure 5F) indicate that VPA, even at a minimal dose, significantly limits glucose metabolism through the PPP and that disruption of the intracellular redox balance through inhibition of G6PD (Figure 5E) and the subsequent inhibition of GR (Figure 5D), GP (Figure 5C) and CAT (Figure 5B) is tightly associated with VPA-induced increases in ROS formation [126,127]. This strongly suggests that the mechanism responsible for the decrease in Na^+^, K^+^-ATPase activity under the conditions used (Figure 4) (in addition to ATP deficiency) may involve a VPA-induced disturbance in redox homeostasis that makes the pump protein vulnerable to oxidative damage [115,117,120]. At the same time, a VPA-induced disturbance in intracellular redox balance will initiate the oxidation of membrane proteins [81] and lipids [82,149], affecting the deformability, rheological behavior and osmotic fragility [141]. This also promotes the formation of macrocytes/stomatocytes [82,140] and the untimely removal of erythrocytes from circulation [150] and other adverse reactions that prevent erythrocytes from performing their normal biological functions.

Furthermore, it was also shown that disrupted redox signaling originating from VPA-induced G6PD inhibition (Figure 5E) and the presence of a higher degree of oxidative stress in the erythrocytes leads to the increased formation of MetHb (Figure 6), which is unable to carry oxygen [151]. Since MetHb had a fast trend (30 min) toward elevation in all the treated animal groups and its level increased 3.5 times in response to higher drug doses of 500–1000 mg/kg, this should be a warning sign at least because the chronic dose-dependent effect of VPA on this parameter indicating inadequate tissue oxygenation is currently unknown.

In parallel with the maximal rise in the MetHb content under the influence of VPA at doses of 500–1000 mg/kg bm, we also found a maximum decrease (almost 2 times as compared to the control) in the level of 2,3-DPG in the erythrocytes of the animals (Figure 7). These data, on the one hand, confirm the accepted opinions about the relationship between oxidative stress, methemoglobinemia and reduced 2,3-DPG levels in the erythrocytes [131]. On the other hand, they indicate that with an increase in MetHb when the G6PD activity is reduced, the remaining hemoglobin develops heightened Hb oxygen affinity (low 2,3-DPG level), which prevents the unhindered release of oxygen from the complex with Hb and its transfer in the required quantity to the tissues and in particular to the brain leading to hypoxia [152,153] and a neurodegenerative process [92].

Surely, apart from 2,3-DPG, there exist other factors such as pH, pCO_2_, pO_2_, Cl^−^, ATP, Mg^2+^, Pi, the conformation and structure of Hb, temperature and so forth that could affect the affinity of Hb to oxygen [154]. However it is acknowledged that 2,3-DPG is the primary biochemical marker of tissue hypoxia [132], associated with the metabolism of erythrocytes.

Overall, the findings suggest that VPA-induced cerebral oxygen depletion [67] and suppression of oxidative metabolism in patients with epileptic seizures [58], resulting in encephalopathy without liver failure [42], may be of erythrocyte origin. VPA-related profound redox imbalance rendering erythrocytes vulnerable to oxidative damage may be one of the main causes of the impairment of the oxygen transport function of erythrocytes leading to encephalopathy.

## 5. Conclusions

In summary, it can be concluded that for further elucidation of complex mechanisms underlying VPA-induced brain hypoperfusion and encephalopathy, it is necessary to measure the parameters of metabolic/glycolytic erythrocyte indicators pointing to the capacity of Hb to bind to oxygen in the lungs and to a release of oxygen in the amount tissues need.

## Data Availability

All data are contained within this article.

## Abbreviations

The following abbreviations are used in this manuscript:

2,3-DPG	2,3-diphosphoglycerate
AN	total adenine nucleotide content
CAT	catalase
CBF	cerebral blood flow
CPS-1	carbamoylphosphate synthetase-1
EnC	energy charge
G6PD	glucose-6-phosphate dehydrogenase
GAPDH	glyceraldehyde-3-phosphate dehydrogenase
GP	glutathione peroxidase
GR	glutathione reductase
GT	glutathione transferase
HA	hyperammonemia
HK	hexokinase
LDH	lactate dehydrogenase
MetHb	methemoglobin
NTB	nitrotetrazolium blue dye
PK	pyruvate kinase
PFK	phosphofructokinase
SOD	superoxide dismutase
VIE	valproate-induced progressive encephalopathy
VPA	Valproic acid
